# Temperature-Mediated Changes in Rates of Predator Forgetting in Woodfrog Tadpoles

**DOI:** 10.1371/journal.pone.0051143

**Published:** 2012-12-12

**Authors:** Maud C. O. Ferrari, Grant E. Brown, Douglas P. Chivers

**Affiliations:** 1 Department of Biomedical Sciences, Western College of Veterinary Medicine, University of Saskatchewan, Saskatoon, SK, Canada; 2 Department of Biology, Concordia University, Montreal, QC, Canada; 3 Department of Biology, University of Saskatchewan, Saskatoon, SK, Canada; University of Regina, Canada

## Abstract

Hundreds of studies have investigated the sources and nature of information that prey gather about their predators and the ways in which prey use this information to mediate their risk of predation. However, relatively little theoretical or empirical work has considered the question of how long information should be maintained and used by prey animals in making behavioural decisions. Here, we tested whether the size of the memory window associated with predator recognition could be affected by an intrinsic factor, such as size and growth rate of the prey. We maintained groups of predator-naive woodfrog, *Lithobates sylvaticus*, tadpoles at different temperatures for 8 days to induce differences in tadpole size. We then conditioned small and large tadpoles to recognize the odour of a predatory tiger salamander, *Ambystoma tigrinum*. Tadpoles were then maintained either on a high or low growth trajectory for another 8 days, after which they were tested for their response to the predator. Our results suggest that the memory window related to predator recognition of tadpoles is determined by both their size and/or growth rate at the time of learning and their subsequent growth rate post-learning.

## Introduction

Whether it relates to foraging, mating or predator avoidance, animals require current and accurate information about their environment to make informed decisions about which behavioural option to pursue [1], [2]. Individuals often rely on indirect cues to assess the quality and suitability of a new habitat. However, as time passes, the value of these cues may decrease, leading to suboptimal decisions. When an individual does not have the possibility of updating the informative value of a given cue, it has to decide whether to use, downplay or ignore older information. Selection should favour individuals able to make optimal use of uncertain information [3], [4], [5].

A number of models have been proposed for adaptive forgetting of information in the context of foraging [1], [6], [7]. The two main ideas coming from these models is that (1) more recent information should have more weight in the decision-making process than older information; and (2), there must exist a memory retrieval window – fixed or variable – that would effectively allow some information to be taken into account in the decision process, while others falling outside the memory window are removed. In these foraging memory models, the rate at which the information value decreases is often linked to the rate at which the environment changes. In constant environments, cues may maintain their reliability for longer, while in highly variable environments, cues may remain informative for only a short period.

While numerous theoretical and empirical studies have examined factors affecting information use and memory in the context of foraging [8], [9], little is known about the effects of such factors in the context of predation. Empirical evidence indicates that different species have different memory retrieval windows for predators. For example, crayfish vary in their retention of learned predators, ranging from 1 day to 4 weeks, depending on the species [10]. Chivers and Smith [11] demonstrated that fathead minnows, *Pimephales promelas*, displayed antipredator responses to the odour of a predator that they learned 2 months before. Juvenile salmonids can retrieve information about their predators for more than 10 days [12] but less than 21 days [13]. Tadpoles of the Iberian green frog, *Pelophylax perezi*, can retain information about predators for up to 9 days [14]. While it is not surprising that different species may possess different memory windows, due to their variability in body and brain sizes, genetic predispositions, or evolutionary history for example, little is known on which factors could affect the length of those memory windows intraspecifically or intra-individually. In one recent study, Ferrari et al. [15] documented that certainty associated with correctly identifying a predator resulted in increasing the length of the memory window. Tadpoles conditioned to recognize a predator 4 times remembered the predator longer than tadpoles conditioned to recognize the predator only once.

Ferrari et al. [16] have identified a number of factors that could potentially affect the length of prey's memory retrieval window, such as body size and growth rate. We predicted that an individual's change in body size results in an internally driven (or intrinsic) change in the informative value of the environmental cues used to assess predation risk. As an individual grows, it may be more or less susceptible to its predators. An individual may become too big for gape-limited predators, or otherwise outgrow them. It may also cease to encounter its predators due to growth-related shifts in macro- or microhabitat use. Alternatively, it may become a prey of choice for other predators. Thus, the size and/or growth rate of a prey may determine how long the information about potential predators should be maintained. To test this hypothesis, we conditioned two size classes of woodfrog tadpoles, *Rana sylvatica*, i.e., on two growth trajectories, to recognize a tiger salamander, *Ambystoma tigrinum*, as a potential predator through simultaneous pairing of injured conspecific cues with salamander odour. A number of aquatic species are known to acquire recognition of predators through this mode of learning [17]. We then maintained the two size classes of tadpoles on either a fast or slow growth trajectory for 8 days before testing them for a response to water or salamander odour. In this system, we predict that an increase in size will result in a decrease in vulnerability, given that tadpoles become better swimmers as they grow [18]. If our hypothesis about growth rate-dependent forgetting of predator recognition is supported, then we predict that tadpoles maintained on a fast-growth trajectory after learning would display a lower response to the predator than tadpoles on a slow-growth trajectory. Our experiment also allows us to assess the effect of growth rate pre-learning by comparing the responses of slow growing (i.e. small) tadpoles versus fast growing (i.e. large) tadpoles. For ease of presentation we refer to the pre-learning treatment as size (small vs large tadpoles learning to recognize the predator) and the post-learning treatment as growth rate (tadpoles maintained on a low vs high growth rate post-learning). We address the size/growth rate dichotomy in the discussion.

## Methods

All work reported herein was conducted in accordance with the Canadian Council on Animal Care and followed the University of Saskatchewan Council on Animal Care and Supply protocol 20060014. None of the work involved the use of protected or endangered species. No specific permits were required for the use of wild aquatic plants. Access to the field site was granted by the land owners.

### Water, predators and test species

Three weeks prior to starting the experiment, a 1900-L tub was filled with well water and seeded with zooplankton, phytoplankton and aquatic plants using a fine mesh dip net. This was done to ensure that our holding and test water did not contain any cues from salamanders. Tiger salamanders occur in the region of our field site in central Alberta, Canada, but our research from the past four years indicates that no salamanders inhabit our study pond and that woodfrog tadpoles do not show any innate recognition of salamander cues [19]. This water is hereafter referred to as well water.

Six tiger salamanders (snout-vent length: mean ± SD = 11.1±0.6 cm) were caught from a pond on the University of Saskatchewan campus in April 2011 using Gee's Improved minnow traps. The salamanders were kept in a plastic tub containing 30 L of well water and fed earthworms every two days.

Woodfrog egg clutches were collected in early May 2011 from a pond in central Alberta. Six clutches laid the same night were transferred into a plastic pool filled with pond water and left floating on the pond to equalize the temperatures of the water in the pool and pond. After hatching, the tadpoles were provided with rabbit chow to supplement the algae already present in the pool. The tadpoles were raised for two weeks before being used.

### Stimulus preparation

Six salamanders were used to prepare the predator odour. Individual salamanders were soaked in 2 L of well water for 24 hr. The odours from two salamanders were then combined and frozen in 200-mL aliquots. Odours from each of the three pairs were randomly used throughout the experiment. The stimulus was thawed and brought to ambient temperature prior to being used. The injured conspecific cue solution used in the conditioning trials was prepared a few minutes prior to being used, by crushing 144 tadpoles with a mortar and pestle in 360 mL of well water. Tadpoles were killed by a blow to the head in sub-groups of 10–15 individuals. The same number of tadpoles from each treatment group was used to prepare cues for conditioning of all treatment groups to ensure that tadpole size would not confound the results.

### Experimental design

**Figure 1:**
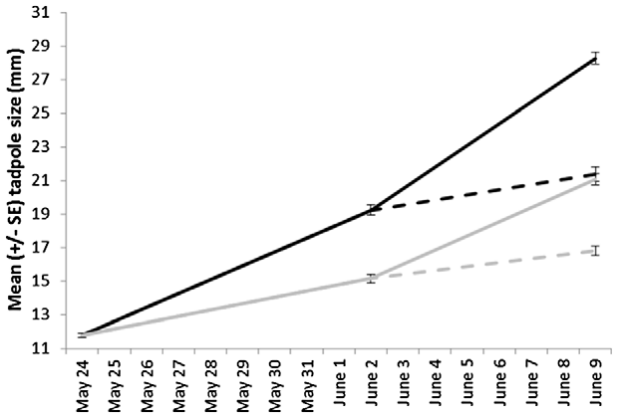
Mean tadpole length measured at the beginning of the experiment (subsample: May 24; N = 60), one day post-conditioning (June 2; N = 18/treatment), and 8 days post-conditioning (June 9; N = 30–36/treatment). Tadpoles were initially maintained under warm (black) or cold (grey) conditions to obtain two distinct size classes. After conditioning tadpoles were maintained under warm temperatures to promote fast growth rate (solid lines) or cold temperatures to promote a slow growth rate (dashed lines).

To obtain two different size classes of tadpoles, we maintained 2-wk old tadpoles in a cold or a warm environment for 7 days. After measuring a subset of the tadpoles and confirming the existence of two distinct size classes (fig. 1), we conditioned them to recognize a tiger salamander as a predator using a one-time conditioning paradigm [17]. Some tadpoles were tested the following day to investigate the effect of size on learned predator recognition. We then maintained half of the remaining small and half of the remaining large tadpoles on a slow-growth trajectory, while the other half was maintained on a high-growth trajectory. The growth trajectory was once again manipulated through temperature. After 7 days, tadpoles were tested for their responses to a water control or salamander odour. Pre-conditioning treatments (warm or cold prior to conditioning) allow us to test the effect of size or growth rate at conditioning on the acquisition and retention of predator recognition. Post-conditioning treatments (warm or cold after conditioning) reflected the post-conditioning effects of the change in the growth rate of the individuals on their retention of predators.

**Step 1: Manipulating pre-conditioning size:** On May 24, 2011, 36 tubs (40×30×30 cm), containing ∼12 L of well water and rabbit chow were set outdoors. One ice pack (15×20 cm) at ambient temperature was placed in each tub. We collected 720 tadpoles from our holding pool, and measured a sub-sample of 60 (total length: mean ± SD = 11.8±0.1 mm). We then arbitrarily placed 20 tadpoles in each of the 36 tubs. Eighteen randomly chosen tubs were assigned to the ‘warm’ treatment while the other half were assigned to the ‘cold’ treatment. On that day, the ice packs from the cold treatment group were replaced by a set of frozen ice packs. Twice a day, around 1200 and 1600 hr, the ice packs from the cold treatment group were replaced with frozen ones. The ice packs from the warm treatment group were removed and put back in the tubs to control for disturbance. This procedure effectively cooled the water from 1200 until ∼1800 hr. Temperature checks were performed 3 times per day, at 1400, 1600 (prior to changing the ice pack) and 1800 hr, to keep track of the actual temperature difference induced by the ice packs. This ‘cooling’ procedure was identical throughout the experiment, both before and after conditioning and testing (temperatures mean ± SD: cold treatment: 11±3°C, warm treatment: 20±4°C). We did not attempt to induce a temperature difference at other times, because the early spring overnight temperatures at our latitude were already low (range 2–10°C).

**Step 2: Conditioning and testing:** Temperature treatment was suspended for this phase, and resumed the day following testing, meaning that all the tadpoles were conditioned and tested at the same temperature (∼15°C). In the morning of Day 7 (1 June), we injected 10 mL of injured conspecific cues paired with 20 mL of salamander odour in each of the 36 tubs. One hr after conditioning, we performed a 100% water change on all the tubs and added rabbit chow. The following day, 4 randomly-chosen tadpoles from each tub were tested for their response to either water (2 tadpoles) or salamander odour (2 tadpoles) using the behavioural assay described below. The size of each tadpole was recorded after testing (fig. 1). These tadpoles were then removed from the experiment.

**Step 3: Post-conditioning temperature manipulation and memory testing:** Temperature treatments resumed the day after testing (day 9). In each of the warm and cold treatment groups, we randomly chose nine tubs that would be maintained in their previous treatment group while the other nine would be exposed to the other treatment group. Thus, we had nine tubs in each of the following group: cold-cold, warm-cold, cold-warm, warm-warm. This phase was conducted in the morning, prior to the frozen ice packs being added. On 9 June, 5 tadpoles from each tub were tested for their response to either water (2 tadpoles) or salamander odour (3 tadpoles) and their length was recorded post testing (fig. 1).

### Testing and behavioural assay

All tadpoles were tested at the same temperature (∼14–16°C). One hr before testing, individual tadpoles were placed in 0.5-L cups filled with well water. Tadpoles were exposed to 5 mL of well water or salamander odour. We used a well-established behavioural protocol to quantify the antipredator responses of tadpoles [19]. We observed the tadpoles for 4 min prior to injecting the stimulus (pre-stimulus period) and 4 min after the injection of the stimulus (post-stimulus injection period). The change in activity between the pre- and post-stimulus periods is interpreted as the behavioural response due to the injection of stimulus. The typical antipredator response of larval amphibians, including woodfrog tadpoles, is to decrease activity upon detection of predation cues. Thus, a line was drawn on the bottom of the testing cups and the number of lines crossed was recorded during the pre and post-stimulus periods. We considered a line was crossed when the entire body of the tadpole was on the other side of the line. The order of testing was randomized between the treatments and the observer was blind to the cues for which tadpoles were tested.

### Statistical analysis

Behavioural analyses were performed to assess the effect of treatment at each testing time. We calculated the change in proportion of line crosses from the pre-stimulus baseline and used those as raw data in our analysis. To account for the dependency of tadpoles originating from the same tub, we added “pail” as a nesting factor in each analysis (“cue” nested within “pail”). Given that tadpoles of different sizes may have differential escape speeds, size is an important factor to consider when comparing the intensity of antipredator responses displayed by tadpoles from different treatments. Linear regressions were used to investigate if the variation in tadpole size would explain any variation in the intensity of their antipredator responses. If it did, the analysis was rerun using the Studentized residuals from the regression as the response variable, to test the effect of treatment without size bias.

To test if size at learning affects the intensity of risk that tadpole learn to associate with the novel predator, we performed a two-way nested ANOVA testing the effect of pre-conditioning size (small vs large) and testing cue (water vs salamander) on tadpole antipredator response one day post-conditioning. To test whether size at conditioning and growth rate post conditioning affect the retention of predator recognition, we performed a three-way nested ANOVA investigating the effects of size at conditioning (small vs large), post-conditioning growth rate (fast vs slow) and testing cues (water vs salamander odour) on tadpole antipredator responses eight days post-conditioning.

## Results

### Testing of tadpoles 1 day after conditioning

**Figure 2:**
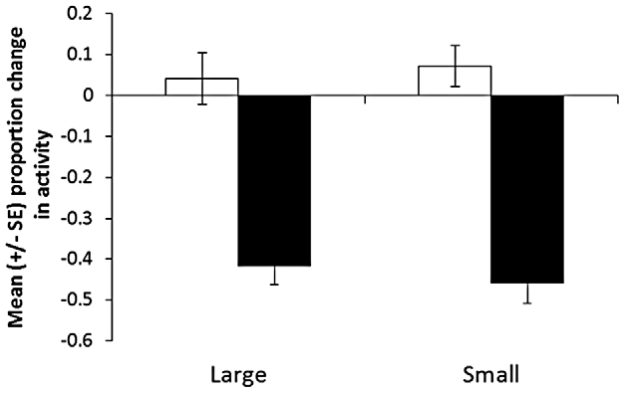
Mean change in activity from the pre-stimulus baseline for large and small tadpoles conditioned to recognize a predatory salamander and tested, one day later, for their response to water (white bars) or salamander odour (black bars). Size classes were obtained by maintaining tadpoles under warm or cold conditions for 7 days prior to conditioning (see text for details).

Our 2-way nested ANOVA revealed that tadpoles responded to salamander odour, but not to water (F_1,34_ = 88, P<0.001, fig. 2). We found no effect of size at conditioning (F_1,34_ = 0.1, P>0.9), no size by cue interaction (F_1,34_ = 0.5, P = 0.5), no effect of pail (F_34,34_ = 0.9, P = 0.6) or cue by pail interaction (F_34,72_ = 1.0, P = 0.5). Linear regressions revealed that individual tadpole size at testing did not explain any significant variation in their responses to any of the cues (water: F_1,70_ = 0.5, P = 0.5; salamander: F_1,70_ = 0.2, P = 0.7).

### Testing of tadpoles 8 days after conditioning

**Figure 3:**
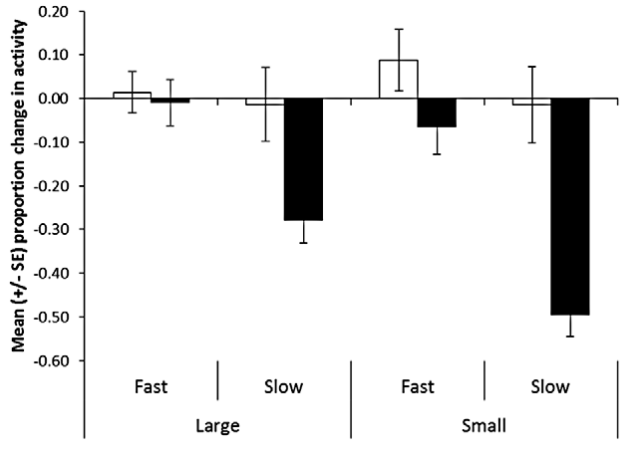
Mean proportion change in activity from the pre-stimulus baseline for large and small tadpoles conditioned to recognize a predatory salamander, maintained on either a fast or slow growth trajectory for 7 days and tested for their response to water (white bars) or salamander odour (black bars).

Our 3-way nested ANOVA revealed a significant interaction between cue and size at conditioning (F_1,31.1_ = 5.8, P = 0.022) and between cue and growth rate (F_1,31.1_ = 15.6, P<0.001, fig. 3). Consequently, we divided the analyses by cue, to further inspect the effect of size and growth rate on the responses of tadpoles to each of the two cues. When looking at the responses of tadpoles to water only, the 2-way ANOVA revealed no effect of size at conditioning (F_1,31.7_ = 0.2, P = 0.6), no effect of growth rate (F_1,31.7_ = 0.7, P = 0.4) and no interaction (F_1,31.7_ = 0.2, P = 0.6) on the responses of tadpoles. Again, pail did not have any affect (F_32,35_ = 1.3, P = 0.2). In addition, size at testing did not explain any variation in the responses of tadpoles to water (linear regression: F_1,69_ = 0.4, P = 0.5).

**Figure 4:**
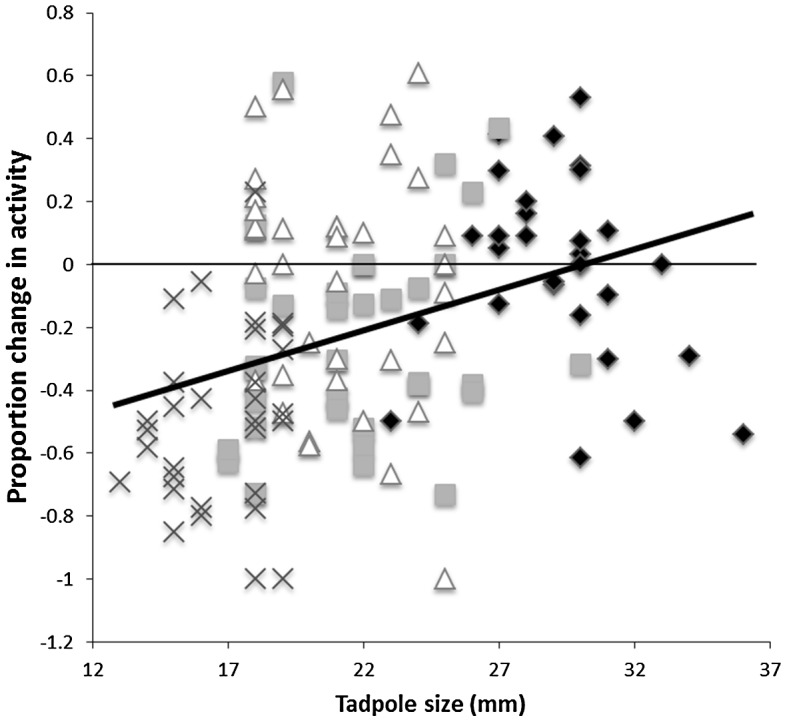
Scatterplot of the proportion change in activity of each tadpole exposed to salamander odour according to tadpole size. The fat solid line represents the regression line (R^2^ = 0.1, P<0.001). Crosses represent small tadpoles maintained on a slow growth rate, triangle small tadpoles maintained on a fast growth rate, square large tadpoles maintained on the slow growth rate and diamonds large tadpoles maintained on a fast growth rate.

**Figure 5:**
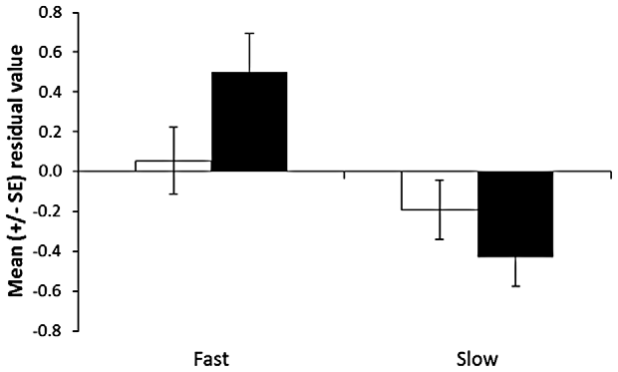
Mean residual value from tadpoles exposed to salamander odour 8 days post conditioning. Large (open bars) and small (black bars) tadpoles were maintained on a fast or slow growth trajectory post-conditioning. Residuals were obtained from a linear regression between tadpoles' intensity of antipredator response when exposed to salamander odour and their size.

When looking at the responses of tadpoles to salamander odour, both size at conditioning (F_1,29.8_ = 4.8, P = 0.036) and growth rate (F_1,30.3_ = 47.9, P<0.001) had an effect on the responses on tadpoles to the predator. Regardless of size, tadpoles on a fast growth trajectory responded less to the salamander than those on a slow growth trajectory. In addition, tadpoles that were small at conditioning responded more to the predator that those that were large. However, we failed to find an interaction between the two factors (F_1,30.4_ = 2.5, P = 0.1). Again, pail did not have an effect on the responses of tadpoles (F_32,95_ = 0.8, P = 0.8). The size of tadpoles at the time of testing did significantly influence the intensity of antipredator response displayed toward the salamander odour (linear regression: F_1,129_ = 18.4, P<0.001, R^2^ = 0.1, fig. 4). To control for this effect, we re-ran the same analysis, using the Studentized residuals from the regression. These data represent the variation in the response of tadpoles to the treatments once the effect of size at testing is removed. When size is taken into account, we found a significant interaction between size at conditioning and growth rate on the responses of tadpoles (F_1,30.5_ = 4.9, P = 0.035, fig. 5). Explicitly, growth rate had on average a stronger effect on smaller than larger tadpoles. Small tadpoles at conditioning finding themselves on a fast-growth trajectory responded the least to the predator, while small tadpoles at conditioning on a slow growth trajectory responded the most. However, growth rate did not seem to affect the response to tadpoles that were large at the time of conditioning.

## Discussion

In this experiment, we used water temperature to manipulate the growth rate of larval amphibians. In warm conditions, tadpoles had high growth rates and reach larger sizes faster than tadpoles maintained in cold conditions. When tested one day post conditioning, we found that the small and large tadpoles did not differ in their response to the salamander. Consequently, the differences in responses among the groups at 8 days post-conditioning reflect differences in retention and not differences in the intensity of learning. Our results suggest that the memory window related to predator recognition of tadpoles is determined by both their size at conditioning and their subsequent growth rate post-conditioning. Those tadpoles that were small at conditioning and those growing slowly post-conditioning, retained the recognition of the salamander as a predator for longer than those that were larger at conditioning and those that were fast-growing post-conditioning. The presence of a significant interaction indicates that the decrease in antipredator response through time is not simply the result of an additive effect of pre and post conditioning conditions. Our results indicate the potential for compensatory forgetting in which smaller tadpoles maintained on the same growth rate as larger ones will forget proportionally faster. These results indicate a dynamic adjustment of the memory window. The duration of the retention of the information is not pre-determined or encoded when the information is acquired but rather changes as the individual grows.

For clarity, we have thus far referred to the pre-conditioning treatment as tadpole size, however, it is likely that growth rate at conditioning and not absolute size per se, was the factor modulating retention. Our data does not allow us to distinguish between these two alternatives. However, Brown et al. [20] documented that pre-learning growth rate and not absolute body size determined how long rainbow trout retained their responses to a learned predator. While we used temperature to manipulate growth rate, we cannot exclude the possibility that lower temperatures could affect the cognitive abilities of tadpoles. Sangha et al. [21] demonstrated that a post-learning 8-day cooling procedure (from 23°C to 4°C) in snails could lead to increased memory capacity. A slower metabolism and reduction in the rate of protein break-down may potentially explain the persistence of memories. In our experiments, the tadpoles were actively cooled for a period of 6 hours per day (noon until 6pm) for 7 days, and the difference in temperature was much smaller than the ones reported above. As here, Sangha et al. [21] could not tease the effect of temperature from those related to growth. Future experiments could attempt to use food as a mean to manipulate growth rate, hence teasing the effects of temperature versus growth.

Empiricists working on information retention in non-human species find it sometimes difficult or even impossible to determine whether the lack of an overt response is the result of a retrieval problem (i.e., the individual has forgotten the information about the predator) or is a result of an active behavioural decision (i.e., the individual recognizes the predator but “decides” not to respond to it). This is a fair criticism for most memory studies. Our design does not allow us to distinguish between these alternatives. Could our results be explained by a pure developmental framework? The concept of infantile amnesia [22] describes a phenomenon in which retention of information could be linked to growth. As a young individual grows, the developing brain undergoes important reorganizational changes, leaving some memories harder to retrieve. Our results certainly fit within this framework. However, Ferrari and Chivers (submitted) have data indicating a difference in memory between tadpoles learning the information for the first time and tadpoles having previously learned and forgotten this information. Regardless of the proximate mechanisms responsible for our observations, ontogenetic dynamic adjustments of memory windows seem particularly adaptive.

In our system, the vulnerability of the tadpoles to predators will likely decrease over time. To decrease their chances of being detected by a predator, many prey species (Lima & Dill 1990), including tadpoles, will initially reduce activity upon detecting that predator. However, if detected, tadpoles have to flee to escape a predator attack. As tadpoles get bigger, and swim faster [18], their probability of escaping predators such as salamanders or predatory diving beetles increases. These observations are likely true for a number of prey species that are growing “out of their predators”. Previous work indicated that maintaining both useless and inaccurate information about predators could be costly [23], [24]. Hirvonen et al. [7] discussed the possibility of a dynamic information devaluation rate, for which the payoff associated with the use of a particular piece of information should determine whether the information is used or ignored in future decisions. Positive payoffs (food rewards) should lead to a higher weighting of the information in future decision-making, while negative payoffs (no food rewards) should lead to the removal of the information. Although intuitively optimal for foragers, this approach would not be suitable for prey, since the first ‘negative payoff’ (failure to respond to a predator) would likely result in the death of the individual. Thus, prey have to devaluate older information in a more graded, risk-aversive manner. Unfortunately, no model currently exists to predict how the specificity of predation-related information would affect optimal forgetting models. More work, both empirical and theoretical, is needed to further understand the factors affecting adaptive information use and how selection pressures, such as predation, affect those traits.
